# Brain Herniation in Neurofibromatosis with Dysplasia of Occipital Bone and Posterior Skull Base

**DOI:** 10.1155/2015/816079

**Published:** 2015-10-27

**Authors:** Vithal Rangarajan, Amit Mahore, Manoj Patil, Prashant Sathe, Amol Kaswa, Sandeep Gore, Pralhad Dharurkar, Juhi Kawale

**Affiliations:** ^1^Department of Neurosurgery, Seth G.S. Medical College and K.E.M. Hospital, Parel, Mumbai 400012, India; ^2^Department of Medicine, Seth G.S. Medical College and K.E.M. Hospital, Parel, Mumbai 400012, India

## Abstract

A 22-year-old female, a known case of neurofibromatosis 1 (NF1), presented with a congenital swelling in the left occipital region. She had developed recent onset dysphagia and localized occipital headache. Neuroradiology revealed a left occipital meningoencephalocele and a left parapharyngeal meningocele. This was associated with ventriculomegaly. She was advised on cranioplasty along with duraplasty which she denied. She agreed to a lumbar-peritoneal shunt. She described a dramatic improvement in her symptoms following the lumbar-peritoneal shunt. Occipital dysplasias, though uncommon, have been reported in the literature. We review this case and its management and discuss relevant literature on occipital dysplasias in NF1.

## 1. Introduction

Neurofibromatosis (NF) type 1 is an autosomal dominant disorder affecting 1 in 2500–3500 individuals. Skeletal dysplasias are well documented in NF1, with scoliosis being the most common. Skull dysplasias do occur but usually restrict themselves to the sphenoorbital region [1]. We describe a rare case of an occipital dysplasia with an accompanying encephalocele.

## 2. Case Report

**Figure 1:**
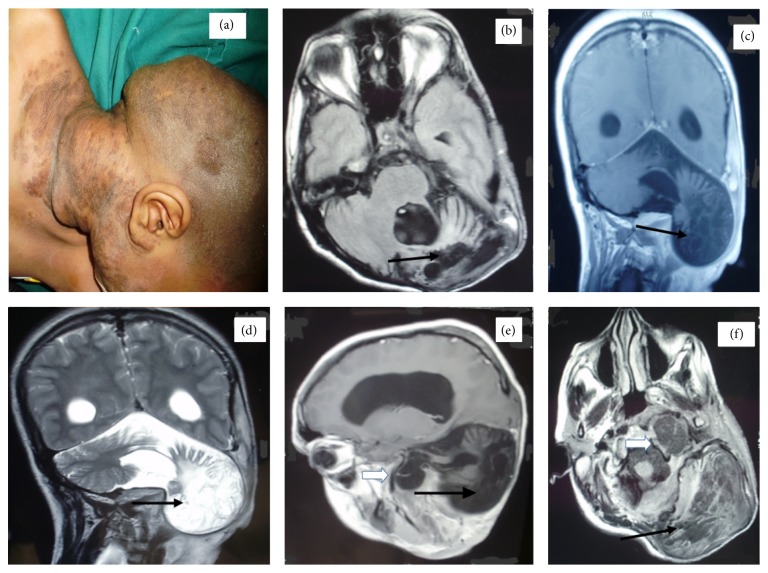
Clinical photo of the plexiform neurofibroma and cafe au lait spots (a). Axial T1 (b, f), coronal T1 (c), coronal T2-weighted (d), and sagittal T1-weighted (e) MR images showing occipital meningoencephalocele (black arrow) and parapharyngeal meningocele (hollow arrow).

A 22-year-old female, with no significant previous medical history, presented with a congenital painless left retroauricular swelling extending into the side of her neck. She consulted us due to recent onset dysphagia and headache. She had left sided cerebellar signs with left sided lower cranial nerve paresis on examination. She also had multiple subcutaneous neurofibromas over her body along with cafe au lait spots and a plexiform neurofibroma (PNF) of the left side of her neck (Figure 1(a)).

Magnetic Resonance (MR) images (Figures 1(b)–1(f)) showed bony dysplasia of the left squamous occipital bone with herniation of the gliotic left cerebellar hemisphere and the tip of the left occipital lobe through the defect into the suboccipital region. Another meningocele was noted extending through the region of the left petrous apex into the left parapharyngeal region. There was dilation of the ventricular system also.

She was advised on duraplasty and cranioplasty along with excision of the redundant scalp. She refused to undergo the suggested procedure; however, she agreed to undergo a lumbar-peritoneal shunt. She had significant improvement in her symptoms as well as a reduction in the local swelling after the procedure. She has no recurrence of symptoms at follow-up of 3 years.

## 3. Discussion

NF1 is the commonest of all the phakomatoses (one in 2000 to 3000 live births), inherited as an autosomal dominant disorder. Sphenoid wing or sphenoorbital dysplasia is one of the diagnostic hallmarks of NF1, occurring in about 5–10% of cases [2]. Cases of temporal and parietal bone dysplasias that occur as an extension of the above have also been reported. However, occipital dysplasias are rarely seen in NF1 [1, 3–5] (Table 1).

Neurofibromatosis is generally considered to be a neurocutaneous disorder with a neural crest origin, with very little emphasis on osseous abnormalities despite osseous dysplasia being one of the seven criteria for diagnosing NF1. However, recent evidence has proven mesodermal involvement in this syndrome with primary or secondary involvement of the skeletal system. Most of the osseous lesions are thought to be secondary to the altered functioning of the NF1 gene. These bony defects develop as a result of progressive mesodermal dysplasia and rarely due to erosion of underlying skull bone caused by neurofibromatosis of scalp [1].

PNF usually have a very indolent course and enlarge gradually. The indications for surgery are pain, cosmetic disfigurement, and neurological deficits. There have been few reports of malignant transformation; however, surgery for PNF, being difficult on account of their high vascularity, is usually not resorted to routinely [1].

In cases of congenital cephaloceles, the bone defect is usually in the occipital bone plus the posterior arches of adjacent cervical spine or less frequently in the occipital bone alone, either superior or inferior to the external occipital protuberance. They can be associated with Chiari II and Chiari III malformation, Dandy Walker malformation, cerebellar dysplasias, diastematomyelia, and Klippel-Feil syndrome. The cerebellum within those cephaloceles is usually dysplastic and gliotic [3].

Neurofibromas are typically of homogeneous low attenuation when compared with adjacent muscle on Computed Tomography (CT) images. The typical MR images of neurofibroma show homogeneous isointensity or mild hyperintensity compared with muscle on T1-weighted images, homogeneous enhancement of the solid component of the tumour after contrast administration, and heterogeneous high signal intensity on T2-weighted images [6].

There are two types of plexiform neurofibromas; one type, the Diffuse Plexiform Neurofibroma, is not “readily demarcated.” The other type is the “nodular plexiform neurofibroma” which is characteristically well-demarcated. Both types are prone to malignant transformation [7].

The treatment for these dysplasias, when symptomatic, would involve repair and reconstruction of the skull and the dural defect. Materials for restoring the bone defect include fascia, autogenous bone, and mesh/plates. If the area of a defect exceeds 3 cm × 3 cm, stereoplasm material should be used. Titanium net should be shaped repeatedly and fixed firmly to meet the physiological structure of the skull and prevent the formation of dead space, which can lead to intracranial infections. Dehydration with pressure dressing is necessary to avoid subcutaneous hydrops [8].

Use of a lumboperitoneal shunt has not been routinely used in such cases. We opted for the procedure on account of the patient refusing the routine procedure. We believe that dysphagia in our patient was due to mechanical pressure by parapharyngeal meningocele and left sided lower cranial nerve paresis; also, the lower cranial nerve paresis might have been caused by mechanical traction on brain-stem and lower cranial nerves due to the cerebellar herniation. The reversal of symptoms after cerebrospinal fluid diversion procedure supports our hypothesis.

## 4. Conclusion

Posterior skull base and occipital bone dysplasia with symptomatic meningoencephalocele may be rarely seen with NF1. Lumbar-peritoneal shunt may be judiciously tried in patients with minimal tumor burden of scalp.

## Figures and Tables

**Table 1:** Previously reported cases of occipital meningoencephalocele in NF1.

Author	Age/sex	Cases	Presentation	Management
Nakasu et al. (1981) [4]	42 yr/M	1	Occipital scalp neurofibroma and underlying bone defect	Excision of scalp tumor with duraplasty and cranioplasty

Renshaw et al. (2003) [5]	54 yr/F	1	Massive plexiform neurofibroma extending from the left occipital region to shoulder area with large left occipital bone dysplasia and extensive cerebellar meningoencephalocele	Resection of the neurofibroma and advancement and rotational skin flaps

Bodhey and Gupta (2006) [3]	28 yr/M	1	Occipital encephalocele with malignant transformation of overlying scalp neurofibroma	Biopsy of the scalp tumour

Dadlani et al. (2013) [1]	17 yr/M	2	Occipital meningoencephalocele with scalp neurofibroma	Duraplasty and cranioplasty
	14 yr/M		Occipital meningoencephalocele with scalp neurofibroma	Subtotal excision of tumour with duraplasty

NF1: neurofibromatosis 1; M: male; F: female.
